# Boosting the acceptance of ^18^F-FET PET for image-guided treatment planning with a multi-centric prospective trial

**DOI:** 10.1007/s00259-023-06426-7

**Published:** 2023-09-08

**Authors:** Karl-Josef Langen, Norbert Galldiks, Philipp Lohmann, Felix M. Mottaghy

**Affiliations:** 1 Institute of Neuroscience and Medicine (INM-3, INM-4), Forschungszentrum Juelich, Juelich, Germany; 2 Department of Nuclear Medicine, RWTH Aachen University Hospital, Pauwelsstraße 30, D-52074 Aachen, Germany; 3 Center of Integrated Oncology (CIO), Universities of Aachen, Bonn, Cologne and Duesseldorf, Germany; 4 Department of Neurology, Faculty of Medicine and University Hospital Cologne, University of Cologne, Cologne, Germany; 5 Department of Radiology and Nuclear Medicine, Maastricht University Medical Center (MUMC+), Maastricht, The Netherlands

PET using radiolabeled amino acids such as O-(2-[^18^F]fluoroethyl)-L-tyrosine ([^18^F]F-FET) has become a valuable diagnostic tool for brain tumours [1]. The Response Assessment in Neuro-oncology Working Group (RANO) has recommended amino acid PET as an additional non-invasive imaging approach to anatomical MRI for glioma diagnostics in both adults and children [2–4] and also for the differentiation of local brain metastases relapse from radiation-induced changes [5]. Despite many publications on the use of ^18^F-FET PET, the tracer is approved for clinical use only in a few countries [6], and there is a lack of prospective multi-centre trials, which is essential for regulatory approval.

With especially the goal to create prospective evidence in a multi-centre setting, the Australian-led Trans-Tasman Radiation Oncology Group (TROG) has planned a study implementing ^18^F-FET PET in glioblastoma (FIG). It is a multi-centre trial (ACTRN12619001735145) designed to establish the role of ^18^F-FET PET in radiotherapy planning and clinical management of patients with glioblastoma. In this issue of the European Journal of Nuclear Medicine and Molecular Imaging, Barry et al. report on the results of nuclear medicine site credentialing in the setting of this study concerning tumour delineation and image interpretation of [^18^F]F-FET PET in the FIG trial [7]. The concept of prospective multi-centre trials on diagnostic or therapeutic radiopharmaceuticals is especially facilitated by Australian authorities and could be a blueprint for Europe. They are an integral part of the medical research landscape, aiming to evaluate the impact of various interventions on different groups of patients and across various healthcare facilities. These trials typically involve collaboration between multiple hospitals, clinics, research institutions, and sometimes even private practices, which is also realized in the study discussed here.

The authors report considerable variation in the recording of quantitative parameters such as the biological tumour volume (BTV) defined as the volume of the pathologically increased amino acid uptake and the maximum and mean tumour-to-brain ratios (TBR_max_ and TBR_mean_) in different centres. This occurred particularly among investigators with little experience in [^18^F]F-FET PET, even though all investigators had detailed instructions on how to perform the analysis. Major violations in BTV determination before radiotherapy were observed in 17% of patients and in 11% of patients in image interpretation at the time of suspected tumour relapse. The interrater variability reported in the study illustrates that the assessment of quantitative parameters derived from amino acid PET may be a significant source of error when conducting clinical trials in multi-centre studies. Consequently, careful training seems necessary even for experienced Nuclear Medicine physicians; alternatively, fully automated segmentation tools might aid towards more reproducible results [8]. These observations are also important for the translation of the method in clinical practice.

Since the FIG study is focused on radiotherapy planning and treatment monitoring of glioblastoma patients, the determination of the BTV is the primary focus of the study. Several biopsy-controlled studies have demonstrated that amino acid PET is able to detect glioma tissue beyond contrast enhancement as well as in non-enhancing tumour portions in contrast to conventional MRI [9–11], which is currently the method of choice to assess the tumour extent for treatment planning. Radiotherapy planning including dose painting based on amino acid PET in newly diagnosed glioma patients has been investigated in several studies and appears to be a safe procedure [2]. To date, there is little data demonstrating prolonged survival after amino acid PET-guided radiation planning or radiation boost [12]. This is probably due to the relatively large safety margin in MRI-guided planning and poor prognosis of the patients in general. Prospective studies in larger patient populations are obviously required to establish the prognostic benefit of amino acid PET-guided radiation planning. To this end, the FIG study is focusing on this important issue and will most likely give a reliable perspective on this question. The multi-centric setting will hopefully allow to gather a reasonable conclusion, taking into account the potential pitfalls on the harmonization and interrater variability.

In addition to its importance for treatment planning, BTV also plays a role in treatment response assessment. Some studies reported that BTV is a sensitive parameter to detect treatment response [13–15]. Therefore, a reliable and standardised determination of BTV is an important prerequisite for the implementation of amino acid PET for the evaluation of response. Of note, the cut-off values of the TBR for tumour volume estimation are based on biopsy-controlled studies in patients with untreated, newly diagnosed gliomas [10]. Therefore, corresponding studies are also necessary in pre-treated patients to verify the validity of this approach.

Although BTV is an important parameter, in clinical routine, it is only used in a smaller proportion of patients. In our clinical setting, the most common indications for the use of [^18^F]F-FET PET are suspected recurrent glioma (46%), unclear brain lesions (20%), treatment monitoring (19%), and suspected recurrent brain metastasis (13%) [6]. In more than 80% of patients, in addition to the visual assessment, clinical decision is based on the parameters TBR_max_ and TBR_mean_. It is of importance to note that in the here discussed FIG study, a better agreement among different centres was observed for these parameters. The median coefficient of variation for TBR_max_ and TBR_mean_ among experts was only about 5%, while it was 21.5% for BTV.

It needs to be considered, however, that especially the parameter TBR_max_ depends on the spatial resolution of the PET scanner and the postprocessing of the image data [16]. Therefore, cut-off values can only be compared between different centres to a limited extent. If [^18^F]F-FET will become a more generally used and accepted tracer validation across centres, harmonization of data analyses comparable to the concepts on [^18^F]F-FDG should be considered.

In summary, the evaluation of BTV seems to be subject to high variability at different centres and requires careful training. In clinical practice, the evaluation of [^18^F]F-FET PET is predominantly based on the parameters TBR_max_ and TBR_mean_, which show less variability between different centres; therefore, it might be worthwhile to consider rather these parameters in future multi-centric settings.

## Data Availability

Not applicable.
